# Characteristics, treatment, and nursing care of patients infected by Sars-CoV-2 hospitalized in intensive care units: multicenter study of colombian hospitals

**DOI:** 10.17533/udea.iee.v40n1e08.

**Published:** 2022-03-29

**Authors:** Olga L. Cortés, María del Pilar Paipa, Carolina Mojica, Yudy A. Rojas, Sandra Pulido, Martha Arias, Maribel Esparza, Natalia Martínez, Skarlet M. Vasquez, Indira Arevalo

**Affiliations:** 1 RN, Ph.D. Email: ocortes@cardioinfantil.org. Corresponding author. Fundación Cardioinfantil Instituto de Cardiología, Bogotá, Colombia. Fundación Cardioinfantil Instituto de Cardiología Bogotá Colombia ocortes@cardioinfantil.org; 2 Nurse, Specialist. Email: mpaipa@cardioinfantil.org Fundación Cardioinfantil Instituto de Cardiología, Bogotá, Colombia. Fundación Cardioinfantil Instituto de Cardiología Bogotá Colombia mpaipa@cardioinfantil.org; 3 Nurse. Email: t-cmojica@cardioinfantil.org Fundación Cardioinfantil Instituto de Cardiología, Bogotá, Colombia. Fundación Cardioinfantil Instituto de Cardiología Bogotá Colombia t-cmojica@cardioinfantil.org; 4 Nurse, Specialist. Email: jrojas@cardioinfantil.org Fundación Cardioinfantil Instituto de Cardiología, Bogotá, Colombia. Fundación Cardioinfantil Instituto de Cardiología Bogotá Colombia jrojas@cardioinfantil.org; 5 Nurse, M.Sc. Email: nursing.liderdp@hus.org.co Hospital Universitario de la Samaritana, Bogotá, Colombia. Hospital Universitario de la Samaritana Bogotá Colombia nursing.liderdp@hus.org.co; 6 Nurse, M.Sc. Email: hospitalizacion.pyp@hus.org.co Hospital Universitario de la Samaritana, Bogotá, Colombia. Hospital Universitario de la Samaritana Bogotá Colombia hospitalizacion.pyp@hus.org.co; 7 Nurse, M.Sc. Email: maribel.esparza@foscal.com.co FOSCAL Clinic, Bucaramanga, Colombia. FOSCAL Clinic Bucaramanga Colombia maribel.esparza@foscal.com.co; 8 Nurse. Email: natalia.martinez@foscal.com.co FOSCAL Clinic, Bucaramanga, Colombia. FOSCAL Clinic Bucaramanga Colombia natalia.martinez@foscal.com.co; 9 Nurse, M.Sc. Autonomous University of Bucaramanga, Colombia. Email: vasquez196@unab.edu.co Universidad Autónoma de Bucaramanga Autonomous University of Bucaramanga Colombia vasquez196@unab.edu.co; 10 Nurse, Master’s student. La Sabana University Clinic, Chia, Colombia. Email: indira.arevalo@clinicaunisabana.edu.co La Sabana University Clinic Chia Colombia indira.arevalo@clinicaunisabana.edu.co

**Keywords:** COVID-19, Critical Care, Nursing Care., COVID-19, Cuidados Críticos, Atención de Enfermería., COVID-19, Cuidados Críticos, Cuidados de Enfermagem

## Abstract

**Objective.:**

To describe the clinical characteristics, treatment, evolution, and nursing care of adult patients with severe acute respiratory distress syndrome who were positive for SARS-CoV-2 and hospitalized in intensive care units (ICUs) during the first peak of the pandemic in Colombia, 2020.

**Methods.:**

Multicenter descriptive study of four high-complexity hospitals in Colombia, which included 473 consecutive adult patients admitted to intensive care units with a confirmed diagnosis of SARS CoV-2. Sociodemographic and clinical information - comorbidities, treatment and evolution - and nursing care provided were included.

**Results.:**

Of the patients included, 43.7% died, 88.8% had pneumonia, and 60.2% developed respiratory distress syndrome. Most of those who died were men. Those who died had a median age of 68.4 years and a higher frequency of comorbidities (hypertension, cardiovascular disease, chronic obstructive pulmonary disease, and higher body mass index). They were admitted to the ICU with higher rate of dyspnea, lower oxygen saturation, and higher score of multiorgan failure. They also more often required mechanical ventilation and pronation therapy and were given more vasopressors and renal replacement therapy.

**Conclusion.:**

People with severe acute respiratory distress syndrome due to COVID-19 who were hospitalized in the ICU had a high risk of death, especially older patients; males; those with cardiovascular, respiratory, and hypertension comorbidities; those who needed mechanical ventilation; and those with an elevated SOFA score. The nursing care of these critically ill patients focused on respiratory care and the prevention of associated complications.

## Resumen

**Objetivo.:**

Describir las características clínicas, tratamiento, evolución y cuidados de enfermería de los pacientes adultos con síndrome de dificultad respiratoria aguda grave, positivos para SARS-CoV-2, hospitalizados en Unidades de Cuidados Intensivos (UCI) durante el primer pico de pandemia en Colombia en 2020.

**Métodos.:**

Estudio descriptivo multicéntrico con la participación de cuatro hospitales de alto nivel de complejidad en Colombia, que incluyó 473 pacientes adultos, admitidos de forma consecutiva en unidades de cuidados intensivos -UCI-, con diagnóstico confirmado para SARS CoV-2. Se incluyó información sociodemográfica, clínica, comorbilidades, tratamiento y evolución, además de los cuidados de enfermería brindados.

**Resultados.:**

Del total de pacientes incluidos fallecieron el 43.7%, presentaron neumonía el 88.8% y un 60.2% desarrollaron síndrome de dificultad respiratoria. Las personas que fallecieron en su mayoría fueron hombres, con una mediana de edad de 68.4 años, con mayor frecuencia de comorbilidades (hipertensión, enfermedad cardiovascular, enfermedad pulmonar obstructiva crónica y mayor índice de masa corporal); además ingresaron a UCI con mayor presencia de disnea, menor saturación de oxígeno, y con puntaje mayor de falla multiorgánica. Así mismo, requirieron con más frecuencia de ventilación mecánica, terapia de pronación, uso de vasopresores y terapia de reemplazo renal. Los cuidados de enfermería de estos pacientes en estado crítico se enfocaron al cuidado respiratorio y la prevención de complicaciones asociadas.

**Conclusión.:**

Las personas con síndrome de dificultad respiratoria aguda grave por COVID-19 que se hospitalizaron en UCI tuvieron un riesgo elevado de fallecer, especialmente los pacientes de mayor edad, sexo masculino y con comorbilidades cardiovasculares, respiratorias e hipertensión arterial, uso de ventilación mecánica y un puntaje puntaje de SOFA elevado. Los cuidados de enfermería de estos pacientes en estado crítico se enfocaron al cuidado respiratorio y la prevención de complicaciones asociadas.

## Resumo

**Objetivo.:**

Descrever as características clínicas, tratamento, evolução e cuidados de enfermagem dos pacientes adultos com síndrome de dificuldade respiratória aguda grave, positivos para SARS-CoV-2, hospitalizados nas Unidades de tratamentos Intensivos (UTI) durante o primeiro pico de pandemia na Colômbia em 2020.

**Métodos.:**

Estudo descritivo multicêntrico com a participação de quatro hospitais de alto nível de complexidade na Colômbia, que incluiu 473 pacientes adultos, admitidos de forma consecutiva nas unidades de tratamentos intensivos -UTI-, com diagnóstico confirmado para SARS CoV-2. Se incluiu informação sociodemográfica, clínica, comorbilidades, tratamento e evolução, além dos cuidados de enfermagens brindados**.**

**Resultados.:**

Do total de pacientes incluídos faleceram 43.7%, apresentaram pneumonia 88.8% e um 60.2% desenvolveram síndrome de dificuldade respiratória. As pessoas que faleceram em sua maioria foram homens, com uma média de idade de 68.4 anos, com maior frequência de comorbilidades (hipertensão, doença cardiovascular, doença pulmonar obstrutiva crónica e maior índice de massa corporal); ademais ingressaram a UTI com maior presença de dispneia, menor saturação de oxigeno, e com pontuação maior de falha multiorgânica. Assim mesmo, requereram com mais frequência de ventilação mecânica, terapia de pronação, uso de vasopressores e terapia de substituição renal. Os cuidados de enfermagem destes pacientes em estado crítico se enfocaram ao cuidado respiratório e a prevenção de complicações associadas.

**Conclusão.:**

As pessoas com síndrome de dificuldade respiratória aguda grave por COVID-19 que se hospitalizaram em UTI tinham um elevado risco de morte, especialmente os pacientes mais velhos; os homens; aqueles com comorbilidades cardiovasculares, respiratórias e hipertensão; aqueles que precisavam de ventilação mecânica; e aqueles com um escore SOFA elevado. Os cuidados de enfermagem destes pacientes criticamente doentes concentraram-se nos cuidados respiratórios e na prevenção de complicações associadas.

## Introduction

In March 2020, the World Health Organization (WHO) announced a new global outbreak of pneumonia caused by a virus belonging to the family of coronaviruses, which was associated with the development of the severe acute respiratory syndrome coronavirus 2 (SARS-CoV-2).(1) This was detected for the first time in Wuhan, China, causing coronavirus disease 2019 (COVID-19), and from its initial detection until January 2022, more than 386.6 million infections worldwide have been confirmed, 306.4 million of which patients recovered and 5.72 million died.(2,3) In Colombia as of January 28, 2022, a total of 5.91 million cases had been confirmed, and a total of 134,781 of those had led to death (5.62 million recovered).(4)

 SARS-CoV-2 infects the lower respiratory tract by binding to angiotensin-converting enzyme 2 (ACE2) in alveolar epithelial cells. This activates an immune response with increased secretion of inflammatory cytokines and chemokines in pulmonary vascular endothelial cells,(5) leading to diffuse alveolar damage with edema and necrosis of alveolar and endothelial cells and a rapid deterioration of oxygenation, which characterizes acute respiratory distress syndrome (ARDS).(6) Patients with more severe disease may develop shock at onset and ARDS requiring admission to the intensive care unit (ICU) for hemodynamic management of shock, mechanical ventilation, antibiotic therapy and other rescue measures.(7-11) The time between the onset of symptoms and the onset of dyspnea has been an average of 5 days, to hospital admission of 7 days, and to the appearance of ARDS of 8 days.(7,8) The particular characteristics of some markers of multiorgan failure show changes in laboratory values, such as lymphopenia and elevated liver enzymes, lactate dehydrogenase, inflammatory markers (e.g., C-reactive protein, ferritin), D-dimer (>1 mcg/mL), procalcitonin, prothrombin time (PT), troponin, and creatine phosphokinase.(7-11)

 Disease progression is observed much more frequently in patients at high risk for severe disease and death, such as in individuals aged ≥ 60 years and patients with comorbidities such as hypertension, cardiovascular disease, chronic respiratory disease, diabetes, and cancer.(8,12) The time between the onset of symptoms and death is estimated at 17.8 days (95% CI = 6.9-19.2), and the time to discharge is 24.7 days (95% CI = 22.9-28.1). In patients confirmed with a laboratory-confirmed diagnosis of COVID-19 in China (n = 70,117 cases), a case fatality rate of 3.67% (95% CI = 3.56-3.80) was estimated. Worldwide, the death rate has been equivalent to 1.4% (95% CI = 0.4-3.5) in patients younger than 60 years and to 4.5% (95% CI = 1.8-11.1) in patients aged 60-65 years or older.(4) The general estimate of the fatality rate for this infection in China was 0.66% (95% CI = 0.39-1.33), which increased with age.(11,13)

Intensive-care nurses provide specialized care and have a high level of risk that is accentuated when caring for patients with COVID-19.(14) The care patterns and care interventions of these professionals are organized according to the needs or problems identified for each patient. Among the different activities are the hourly evaluation of the general state of each patient and the structuring of changes to the care plan according to individual needs. The interventions must be more complex in patients with ARDS-COVID-19 and are oriented toward improving the ventilation/perfusion ratio through the use and control of parameters in mechanical ventilation and the implementation of interventions such as prone position and delivering related care when oxygenation with extracorporeal therapy is needed. Other interventions are designed to reduce complications, improve well-being, and improve patient survival.(14)

This study aimed to describe the clinical characteristics, treatment, evolution, and specific nursing care of adult patients who died and did not die from severe or critical ARDS who were positive for SARS-CoV-2 and were hospitalized in ICUs of hospitals and clinics of Bogotá, Colombia, to support and rethink the appropriate strategies for the mitigation and care of this disease in Colombia and across the world.

## Methods

 This is a longitudinal, multicenter study of a case series of adult patients diagnosed hospitalized with COVID-19. The design had a retrospective component, given that the inclusion of patients began on March 1, 2020, corresponding to the period in which the outbreak began in Colombia, and a prospective component because all the cases admitted to the ICU observed until December 15, 2020 were included. Four hospitals of level IV complexity in Cundinamarca and Santander agreed to participate in the study after evaluation and acceptance of the project by their respective ethics committees.

The study entry criteria included adults aged 18 years or older with a definite positive diagnosis of ARDS due to SARS-CoV-2 obtained by the polymerase chain reaction (PCR) test of a nasopharyngeal sample, who required admission to the ICU due to their severe or critical condition or other existing evidence. Patients who were not treated in the ICU for some reason or who died before admission or in the first 4-6 hours in the ICU were excluded. All hospitals included about 25% of the participants. No sample size calculation was performed.

We defined a severe case as the presence of oxygen saturation (≤92%) at rest or PaO2/F_IO2_ of <300 mmHg. Critical cases included patients with a diagnosis of respiratory failure, shock, or multiorgan failure who required mechanical ventilation. The severity was judged by the Berlin classification of ARDS.(15) The inclusion of at least 150 patients in the participating centers was planned before the signing of informed consent [administered in each ICU to family members]. Follow-up was performed from the time of admission of all consecutive patients to the ICU until discharge from the ICU due to recovery, hospital discharge, or death. The main outcome included the clinical outcome of alive vs. deceased. Living was defined as surviving at the time of discharge from the ICU. Death was recorded from a diagnosis of death by the attending physician.

Other variables measured were (i) sociodemographic aspects; (ii) health history [comorbidities, body mass index], previous treatments, and consumption of toxic substances [cigarettes, alcohol, stimulants]; (iii) epidemiological data related to the onset of COVID-19 (probable contagion, time of onset of symptoms, symptoms at the time of admission, length of stay in the ICU, length of stay in the hospital); (iv) classification of severity and medical treatment; (v) variables related to support/rescue procedures (use of extracorporeal membrane oxygenation or renal replacement therapy); and (vi) nursing care related to the prone position, onset time after the start of invasive ventilation, initiation of early mobilization, and care related to the well-being of the patient. The leaders of each center received training on the inclusion of patients and collection of information. They also received training on the inclusion of information in the study platform [http://investigacionenfermeriauci.org/], in which the identification of each center was anonymized.

The statistical analysis plan included the proportion of patients admitted to the ICU out of the total number of infected patients treated in each center and the proportion with each severity of ARDS. The characteristics, interventions, procedures, and nursing care of the individual patients in the ICU are reported as count (%), mean (standard deviation, SD), and median (interquartile range) for deceased and alive patients. Similarly, the time ranges (hours) between onset of symptoms and admission to the ICU, start of mechanical ventilation, and death were described, as were the time of admission to the ICU and initiation of the prone position, length of stay in the ICU, length of hospital stay, discharge time, and confirmation of tests in suspected patients. To compare categorical variables between groups (living and deceased), we used the chi-squared test or Fisher's exact test. For continuous variables, we used Student’s t test or the Mann-Whitney-Wilcoxon test.

## Results

By December 2020, 4426 patients had been admitted to the four hospitals participating in the study, with a diagnosis of pneumonia and a confirmation for SARS-CoV-2. Of these patients, 587 (13%) were admitted to the ICU and were critically ill, of whom 476 (82%) were included in the present study. The other 111 patients were not included since neither the patients nor their relatives had the opportunity to sign an informed consent form. A total of 207 (43.7%) enrollees died, and 266 (56.3%) survived.

The majority of the patients (70%) were men, with a median age of 63.6 years, were from urban regions (93.2%), came from low socioeconomic strata (54.6%), were contributory regime members (82.2%), did not live alone (83.7%), and had a poor educational level (68.3%). 56.3 percent lived and 43.7 percent died. With a median age of 68.4 years, there was a substantial difference in prevalence, with a higher frequency in non-living persons (Table 1).

A higher frequency of arterial hypertension (HTN), obesity, cardiac disease, and current cigarette smoking were identified among the comorbidities discovered (Table 2).

Among the observed antecedents, those who died had a higher incidence of comorbidities (statistically significant) than those who lived. Hypertension, chronic obstructive pulmonary disease-COPD-, cardiomyopathies, arrhythmias, and peripheral vascular disease were more common in the deceased. Similarly, the deceased had a greater body mass index-BMI- than the living.

**Table 1:** Sociodemographic characteristics of 473 patients included in the study according to vital status at ICU discharge**.**

Characteristics	**Total *n*=473**	**Deceased *n*=207**	**Alive *n*=266**	***p-v*alue**
Male sex, n (%)	331 (70.0)	144 (69.6)	187 (70.3)	0.863
Age years, median (IQR)	63.6 (53.4-73.1)	68.4 (60.5-75.7)	59.3 (47.7-68.9)	<0.001
Urban area of origin [*n*= 455], n (%)	424 (93.2)	178 (89.5)	246 (96.1)	0.008*
Socioeconomic stratum [*n*=365], n (%)				0.643*
1-2	199 (54.6)	92 (57.1)	107 (52.4)	
3-4	160 (43.8)	66 (41.0)	94 (46.1)	
5-6	6 (1.6)	3 (1.9)	3 (1.5)	
Social security regime [*n*=460], n (%)				0.117*
Contributive	378 (82.2)	157 (77.7)	221 (85.7)	
Subsidized	60 (13.0)	33(16.3)	27 (10.5)	
Linked/uninsured	13 (2.8)	6 (3.0)	7 (2.7)	
Living arrangement [*n*=429], n (%)				0.335
Not alone	359 (83.7)	157 (81.8)	202 (85.2)	
Alone	70 (16.3)	35 (18.2)	35 (14.8)	
Occupation [*n*=361], n (%)				0.010*
Housewife	90 (24.9)	43 (29.2)	47 (22.0)	
Employee	88 (24.4)	30 (20.4)	58 (27.1)	
Retired	80 (22.2)	41 (27.9)	39 (18.2)	
Health worker	11 (3.0)	1 (0.7)	10 (4.7)	
Independent worker	92 (25.5)	32 (21.8)	60 (28.0)	
Schooling level [*n*=224], n (%)				0.481*
Primary	86 (38.4)	34 (44.7)	52 (35.1)	
Baccalaureate	67 (29.9)	19 (25.0)	48 (32.4)	
Technician	16 (7.1)	6 (7.9)	10 (6.8)	
University	55 (24.6)	17 (22.4)	38 (25.7)	

IQR = interquartile range, * Fisher's exact test

**Table 2:** Health history of the 473 patients according to vital status at ICU discharge**.**

Characteristics	**Total *n*=473**	**Deceased *n*=207**	**Alive *n*=266**	***p-*value**
Comorbidities and Habits				
Arterial hypertension [*n*=471], *n* (%)	243 (51.6)	125 (61.0)	118 (44.4)	<0.001
Obesity [*n*=451], *n* (%)	119 (26.4)	54 (27.5)	65 (25.5)	0.623
BMI [*n*=451], median (IQR)	27.1 (24.2-30.3)	27.3 (24.4-30.4)	26.7 (23.9-30.3)	0.003†
COPD [*n*=466], *n* (%)	31 (6.7)	21 (10.4)	10 (3.8)	0.004
Cardiopathy [*n*=467], n (%)	46 (9.9)	29 (14.2)	17 (6.5)	0.005
Renal disease, n (%)	36 (7.6)	19 (9.2)	17 (6.4)	0.257
Vascular peripheral disease [*n*=467], n (%)	23 (4.9)	15 (7.3)	8 (3.1)	0.050*
Active cancer [*n*=466], n (%)	21 (4.5)	12 (5.9)	9 (3.4)	0.260
Arrhythmias [*n*=466], n (%)	20 (4.3)	14 (6.9)	6 (2.3)	0.020
HIV/autoimmune illness [*n*=465], n (%)	13 (2.8)	7 (3.5)	6 (2.3)	0.573*
Stroke *n*=463, n (%)	8 (1.7)	3 (1.5)	5 (1.9)	1.000*
Currently pregnant *n*=107, n (%)	3 (2.8)	1 (2.2)	2 (3.2)	1.000*
Smoker [*n*=442], n (%)	36 (8.1)	15 (7.8)	21 (8.4)	0.801
Alcohol drinker [*n*=439], n (%)	15 (3.4)	11 (5.8)	4 (1.6)	0.031*
**Pharmacological history**, *n* (%)				
Antihypertensives [*n*=470]	228 (48.5)	115 (56.1)	113 (42.6)	0.004
Beta blockers [*n*=466]	82 (17.6)	54 (26.5)	28 (10.7)	<0.001
Lipid-lowering drugs [*n*=460]	74 (16.1)	42 (21.0)	32 (12.3)	0.012
Diuretics [*n*=463]	64 (13.8)	39 (19.3)	25 (9.6)	0.003
Anticoagulants [*n*=463]	50 (10.8)	32 (15.9)	18 (6.9)	0.002
Pregnancy history [*n*=141], n (%)	56 (39.7)	21 (33.3)	35 (44.9)	0.164

* Fisher's exact test. COPD = chronic obstructive pulmonary disease, HIV = human immunodeficiency virus, BMI = body mass index, IQR = interquartile range, SD = standard deviation.

The most frequent symptoms seen at the time of admission were dyspnea, cough, fever, and fatigue. Significant differences were observed, with a greater presence of dyspnea, fatigue, and nausea in those who died. (Table 3). The median time between the onset of symptoms and hospital admission was 7 days in both groups. The median time between hospital admission and admission to the ICU was 3.5 days (Table 4), which was also similar between groups (Table 3).

**Table 3:** Signs and symptoms at the time of diagnosis of COVID-19 of the 473 patients included in the study according to vital status at ICU discharge**.**

Characteristics	**Total *n*=473**	**Deceased No *n*=207**	**Alive *n*=266**	***p*-value**
Fever [*n*=469], n (%)	318 (67.8)	130 (63.4)	188 (71.2)	0.073
Cough [*n=*471], n (%)	373 (79.2)	169 (82)	204 (77)	0.180
Fatigue [*n*=467], n (%)	227 (48.6)	115 (56.4)	112 (42.6)	0.003
Dyspnea [*n*=472], n (%)	374 (79.2)	174 (84.5)	200 (75.2)	0.014
Nausea [*n*=466], n (%)	44 (9.4)	28 (13.7)	16 (6.1)	0.006
Myalgia [*n*=467], n (%)	169 (36.2)	80 (39.6)	89 (33.6)	0.180
Diarrhea [*n*=464], n (%)	78 (16.8)	38 (18.7)	40 (15.3)	0.332
Pain swallowing [*n*=468], n (%)	144 (30.8)	61 (29.9)	83 (31.4)	0.721
Other symptoms [*n*=466], n (%)	282 (60.5)	133 (64.7)	149 (57.3)	0.112
Time between onset of symptoms and hospital admission, median (IQR), days	7 (4-10)	7 (4-9)	7 (4-10)	0.239

IQR = interquartile range.

The most common diagnosis of ICU admission was pneumonia (413 patients, 88.8%), of which 271 (60.2%) cases advanced to ARDS and 58 (12.9%) were accompanied by shock at admission. Severe ARDS was more prevalent in deceased patients than in living patients (Table 4). Among the risk indicators at admission of the patients who died were lower hemodynamic and ventilatory parameters, such as systolic and diastolic blood pressure. Upon admission to the ICU, those who died had a lower oxygen saturation, with higher ventilatory support parameter requirements of tidal volume, than the living patients. Likewise, the deceased had a higher Sequential Organ Failure Assessment (SOFA) score.

Table 5 lists the nursing treatments administered. The patients who died required greater support with mechanical ventilation, with a longer dwell time, than the living patients (*p*<0.001). Of all the patients, 59.8% received pronation, and this rate was higher in those who died (78.7%). The medications that were administered most often were antibiotics, anticoagulants, dexamethasone, vasopressors, inotropes, and bronchodilators. Of these medications, only bronchodilators and dexamethasone were given at similar rates between the two groups. Additionally, those who died required the insertion of central catheters, peripherally inserted central catheters (PICCs), rescue therapy with renal replacement, and the start of enteral nutrition (EN) more frequently for their care in the ICU than the live group (all *p*<0.05).

**Table 4:** Characteristics related to ICU admission of patients included in the study according to vital status at ICU discharge**.**

Characteristics	**Total n=473**	Deceased n=207	**Alive n=266**	***p*-value**
Admission diagnosis, n (%)				
Pneumonia [*n*=465]	413 (88.8)	185 (90.7)	228 (87.4)	0.258
ARDS [*n*=450]	271 (60.2)	121 (61.1)	150 (59.5)	0.733
Shock [*n*=451]	58 (12.9)	45 (22.3)	13 (5.2)	<0.001
**Place of referral, n (%)**				
Emergency room	247 (53.5)	121 (59.6)	126 (48.7)	0.019
Hospitalization	187 (40.5)	70 (34.6)	117 (45.0)	0.025
Time between hospital admission and admission to the ICU, median (IQR), days	3.5 (1-4)	3.4 (1-4)	3.6 (1-4)	0.979
**Degree of severity of ARDS** , n (%)				0.139
Mild ARDS	14 (5.3)	6 (5.1)	6 (5.4)	
Moderate ARDS	60 (22.6)	20 (17)	40 (27)	
Severe ARDS	192 (72.2)	92 (78)	100 (65.6)	
**Hemodynamic variables, median (IQR)**				
Systolic BP mmHg	120 (107-134.5)	117 (101-133)	124.5 (111-135)	0.005
Diastolic BP mmHg	74 (61-82)	70 (59-81)	74 (64-84)	0.008
Temperature °C	36.2 (36-37)	36.3 (36-37)	36.2 (36-37)	0.879
Heart rate (beats/min)	87 (74-100)	86 (72-101)	87 (75-99)	0.839
Respiratory rate, min	22 (18-26)	22 (18-28)	22 (18-25)	0.297
Oxygen saturation, mean (SD)	89.7 (7.3)	88.8 (7.5)	90.4 (7.1)	0.024
F_iO2_ (%)	70 (35-90)	70 (50-90)	60 (32-90)	<0.001
SOFA score [*n*=339]	6 (3-10)	8 (4-11)	5 (3-8)	<0.001
Barthel score [*n*=132]	100 (52-100)	100 (80-100)	100 (47-100)	0.290
Delirium CAM-ICU [*n*=267], n (%)	11 (4.1)	1 (1.1)	10 (5.8)	0.103*
**Parameters at the beginning of ventilatory support)**				
Oxygen saturation% [n= 347]	92 (89-94)	92 (88-94)	92 (90-94)	0.224
F_iO2_% [*n*=341], median (DS)	75.5 (25.1)	76 (25.1)	74.9 (25.3)	0.893
PaO_2_ [*n*=294], median (RIC)	61 (51-75.9)	58 (49-70)	65 (57-84.6)	<0.001
PEEP [*n*=342], media (DS)	11.3 (2.6)	11.2 (2.8)	11.3 (2.2)	0.357
Tidal volume [*n*= 132], median (IQR)	430 (400-480)	440 (408-480)	387 (420-480)	0.189
**Laboratories, median (IQR)**				
Troponin I [*n*=271]	0.02 (0.01-0.208)	0.04 (0.01-0.69)	0.01 (0.01-0.09)	<0.001
Creatinine [*n*=462], mg/dl	0.9 (0.76-1.17)	0.97 (0.8-1.34)	0.9 (0.7-1.1)	<0.001
Secondary prothrombin time [*n*=418]	14.1 (12.2-15.1)	14.1 (11.7-15.8)	14.2 (12.3-15.4)	0.688
Secondary partial thromboplastin time [*n*=415]	30.1 (28.5-32.4)	30.5 (28.7-34)	30.1 (28.4-31.5)	0.044
PCR mg/L [*n*=397]	39.5 (10.3-150.8)	79.1 (16-169.4)	27.4 (6.2-138.3)	<0.001
High-flow oxygen devices [*n*= 347], n (%)				0.225
Non-reinhalation mask	238 (68.6)	116 (64.8)	122 (72.6)	
Orotracheal tube	91 (26.2)	54 (30.2)	37 (22)	
Venturi	18 (5.2)	9 (5)	9 (5.4)	

ARDS = acute respiratory distress syndrome, BP = blood pressure, IQR = interquartile range. * Fisher's exact test

**Table 5:** Treatment during the ICU stay of the 473 patients included in the study according to vital status at ICU discharge.

Nursing care	**Total** ***n*=473**	**Deceased** ***n*=207**	**Alive** ***n*=266**	***p*-value**
**Type of ventilation, n (%)**				<0.001
Invasive (mechanical ventilation)	348 (73.6)	202 (97.6)	146 (54.9)	
Noninvasive	16 (3.4)	1 (0.5)	15 (5.6)	
Days between admission and start of mechanical ventilation, median (IQR)	3 (1-6)	3 (2-7)	3 (1-5)	0.063
Days on ventilation, median (IQR)	12 (7-18)	13 (8-18)	10 (6-18)	0.045
**Pronation, n (%)**	283 (59.8)	163 (78.7)	120 (45.1)	<0.001
Number of hours/day in pronation *n*= 275, median (IQR)	16 (16-24)	16 (16-24)	16 (16-18)	0.003
**Administration of medications, n (%)**				
Antibiotics	447 (94.5)	201 (97.1)	246 (92.5)	0.029
Dexamethasone	396 (84.3)	177 (86.3)	219 (82.6)	0.275
Vasopressors	246 (52.3)	175 (84.5)	71 (27)	<0.001
Antivirals	26 (5.6)	15 (7.5)	11 (4.2)	0.136
Antimalarials	21 (4.6)	9 (4.6)	12 (4.6)	1.000*
Bronchodilators	240 (51.6)	102 (50.7)	138 (52.3)	0.744
Inotropics	178 (38.4)	127 (62.6)	51 (19.6)	<0.001
Anticoagulants	407 (86.2)	186 (90.3)	221 (83.1)	0.024
**Drug infusion catheter, n (%)**				
Central catheter	212 (45.3)	132 (64.4)	80 (30.4)	<0.001
PICC	204 (43.8)	101 (50)	103 (39)	0.018
Peripheral catheter	281 (59.9)	107 (52.2)	174 (65.9)	0.003
				
**Extracorporeal membrane for oxygenation** , n (%)	3 (0.7)	1 (0.5)	2 (0.8)	1.000
**Renal replacement therapy , n (%)**	79 (16.9)	61 (29.8)	18 (6.8)	<0.001
**Enteral nutrition catheter , n (%)**	338 (71.9)	190 (92.7)	148 (55.9)	<0.001
**Parenteral nutrition catheter , n (%)**	15 (3.2)	7 (3.4)	8 (3.0)	0.799*

IQR = Interquartile range, TOT = Orotracheal tube.

* Fisher's exact test

In average, 83.6 percent of patients received bed position alterations as part of the nonpharmacological therapy (Table 6) offered by nurses. Patients who died had more positional changes than those who were alive (89.8% vs. 78.9%, p=0.002). Oral hygiene was advocated more frequently in surviving patients (92.4 vs. 83.4, p=0.002) than in deceased patients. Communication between the patient and his or her family (by phone or video call) was more common in those who survived than in those who died.

**Table 6:** Nursing care

Nursing care	**Total** ***n*=473**	**Deceased** ***n*=207**	**Alive** ***n*=266**	***p*-value r**
Skin care measures				
Changes in bed position [*n*= 470], n (%)	393 (83.6)	184 (89.8)	209 (78.9)	0.002
Number of position changes/day [*n*= 366], mean (IQR)	2.6 (2-2)	2.7 (2-2)	2.6 (2-2)	
Promotion of early mobilization, n (%)	95 (20.1)	62 (30.0)	33 (12.4)	<0.001
**Comfort Measures**				
Bed bath, n (%)	459 (97.04)	201 (97.1)	258 (97.0)	
Number of bed baths/day [n= 453], median (IQR)	1 (1-2)	1 (1-2)	1 (1-2)	
**Oral hygiene,** n (%)	416 (88.5)	171 (83.4)	245 (92.4)	0.002
**Promotion of communication with family**				
Telephone [*n*=367], n (%)	234 (63.8)	109 (60.9)	125 (66.5)	
Video call [*n*=365], n (%)	191 (52.3)	82 (45.8)	109 (58.6)	0.014
In person [*n*=366], n (%)	18 (4.9)	11 (6.2)	7 (3.7)	

IQR = interquartile range.

The hospital stay was 18 days in general (IQR 11-26), being longer in living patients than in deceased patients (19, IQR 11-29 vs. 17, IQR 11-22, *p* = 0.010). The median ICU stay was 12 days (IQR 6-19) and was also longer in the deceased (median 15, IQR 9-20 vs. 9, *IQR* 4-17, p≤0.001). Complications in the ICU are shown in Table 7. Among the most frequently identified in deceased patients compared to living patients were septic shock (63.3 vs. 12.4*, p<*0.001), renal failure (47.8 vs. 8.3, *p*<0.001), and thromboembolism (13.0 vs. 6.4, *p=*0.006). Pressure ulcers were also more frequent in deceased patients than living patients (38.7 vs. 15.8, *p<*0.001).

**Table 7:** Complications during hospitalization of patients included in the study according to vital status at ICU discharge**.**

Complications n (%))	**Total** ***n*=473**	**Deceased** ***n*=207**	**Alive** ***n*=266**	***p-*value**
Septic shock	164 (34.7)	131 (63.3)	33 (12.4)	<0.001
Renal failure	121 (25.6)	99 (47.8)	22 (8.3)	<0.001*
Pressure ulcers	122 (25.8)	80 (38.7)	42 (15.8)	<0.001
Arrhythmias	72 (15.2)	57 (27.5)	15 (5.6)	<0.001
Delirium	63 (13.3)	16 (7.7)	47 (17.7)	0.006
Thromboembolism	44 (9.3)	27 (13)	17 (6.4)	0.006
Acute myocardial infarction	17 (3.6)	14 (6.8)	3 (1.1)	0.002*
Myocarditis	9 (1.9)	9 (4.3)	-	<0.001*
Intravascular coagulation	7 (1.5)	6 (2.9)	1 (0.4)	0.080

* Fisher's exact test

## Discussion

 We report in our study 473 critically ill patients with a confirmed diagnosis of SARS-CoV-2 acquired during the first outbreak of the pandemic in Colombia in 2020 who were hospitalized in intensive care units in the four participating hospitals. A total of 207 patients died (43.8%), and 266 (56.8%) survived. Most were admitted with a diagnosis of pneumonia (88.8%)and with ARDS (60.2%). Multiple studies have reported the characteristics and evolution of patients hospitalized for COVID-19 in intensive care units since the beginning of the pandemic around the world. We find some similarities and differences in relation to these references, which we present below. Our paper also includes descriptive information related to nursing care in critical patients.

The mortality rate of 43.7% was very similar to that reported by the Intensive Care National Audit & Research Center (ICNARC) in infected patients in 2020.(16) This report included a mortality of 48.6% of a total of 224,748 admitted to 263 ICUs (London, UK). However, the length of stay in the ICUs of our study was longer in those who died (median 15 days) than those who survived (median 9 days), contrary to that reported by the ICNARC ICUs, in which the length of stay of the deceased was approximately 8 days and of those who survived 13 days.(16)

Characteristics such as age, sex, and having some comorbidities have been associated with greater susceptibility to infection and complications after infection by SARS-CoV-2.(7-11,17) In our data, the majority of those infected by SARS-CoV-2 who died were men, and they were older than the survivors, consistent other studies.(7,8) The identification of comorbidities has been key in the characterization of those who may be at risk of lower survival. In our study, the results coincide with those reported in a sample of 46,248 patients admitted to the ICU with a diagnosis of COVID-19, included in a systematic review of eight studies reported by Yang *et al.*(18) They found higher mortality in patients with a history of cardiovascular disease [OR 3.42], respiratory system disease [OR 2.46], or arterial hypertension [OR 2.46]. Overweight in our study showed a higher frequency in the deceased (not significant), similar to the findings of Yang et al*.* (27.3 vs. 26.7, *p*=0.187).(18)

The most frequent symptoms at the time of admission were cough (79%), dyspnea (79.2%), fever (67.8%), and fatigue (48.6). Dyspnea and fatigue were more frequent in patients who died than those who did not. These data are similar to the data reported, for example, in the systematic review and meta-analysis conducted by Grant *et al.,*(19) which included 138 studies conducted in nine countries. In that study, the most prevalent symptoms were fever (78%) and fatigue (31%).(19) Nausea was frequent in our cohort but had a very low prevalence in the study by Grant *et al.* (19)

Considering that patients with COVID-19 often have bilateral interstitial pneumonia/ARDS with acute hypoxemic respiratory failure and multiple-organ failure, we observed some indicators that have been reported as determinants of prognosis in these patients upon admission to the ICU. Among the predictors of mortality, we observed that at the time of admission, patients who died in the ICU had a higher SOFA score, consistent with Gao et al., in which a high SOFA score was associated with a high risk of mortality (HR 1.171, 95% CI 1.013-1.354, *p* = 0.033). (17) In this same group, another indicator was the need for invasive mechanical ventilation, more pronation being required in more severe disease. Respiratory nursing care related to the adequate administration of mechanical ventilation parameters and the implementation of pronation are some of the most frequent interventions in the ICU, especially in patients diagnosed with ARDS due to COVID-19. We found elevated mortality in patients exposed to mechanical ventilation, 58%, higher than the 41% reported in several studies during the first peak of the pandemic. Medications are administered hourly and according to the medical prescription by the nursing professional through a central catheter or PICC.(20) For the most part, the insertion of the PICC, insertion of peripheral lines, and care during the administration of medications is performed by the nursing professional, a constant of quality in the ICU.(20)^.^ Specialized medications are given by nurses according to the critical state of each patient and the system of greatest compromise (cardiovascular, renal, neurological, immunological, and systemic).

Among the drugs administered during the first outbreak in our country, two drugs, the antiviral remdesivir(21) and the immunomodulatory corticosteroid dexamethasone (RECOVERY Trial Study),(22) were approved for use in these patients. The evidence suggests they help manage and reduce the mortality of those who require respiratory support with oxygen or mechanical ventilation. During the first peak of the pandemic, we observed the beginning of the management of the disease with antivirals with a very low frequency of use, which makes it difficult to establish an estimate of their effect. The frequency of use of dexamethasone in these patients was very similar in both the living and the deceased. Anticoagulants were also given to 86.2% of these patients with COVID-19 as part of its treatment to prevent the occurrence of thromboembolic events and their possible complications, as was mentioned in some studies.(23)

 In our study, cardiovascular support with inotropes and vasopressors was used mostly in patients who died in the ICU, given the severity of the disease and the presence of complications such as septic shock and renal failure. The most frequent complication in our study was septic shock, whose association with mortality in COVID-19 patients has also been similar in other studies (OR of 3.2, *p* = 0.003).(24) Similarly, support with renal replacement therapy was required in 17% of critically ill patients with acute kidney injury. Renal failure was observed much more frequently in the deceased, in line with the report of the study by Ferrando *et al.,*(24) which showed an increased probability of mortality in patients with renal failure and COVID-19 (OR of 2.4). These results are similar to the ICNARC report,(16) which showed that 26.7% of patients in the final state in the ICU required advanced renal support, 65% basic cardiovascular support, and 30.4% advanced cardiovascular support.

Other nursing care described was nutritional support, preventive care for the onset of delirium, skin care, and basic care such as body and oral hygiene. Regarding nutritional support in our study, of the total number of patients included in the ICU who received EN, 72% had been reported in similar studies.(25) The deceased received this intervention in greater proportion, but it is not possible to establish any association with EN, since we do not have information on the onset of EN, the status or nutritional assessment of the patients at this time, or other associated complications of pronation, which can determine the relationship between survival and EN use.

 The nursing activities aiming to promote behaviors that improve the COVID-19 patient’s mental orientation (prevention of delirium), calmness, and cooperation in the ICU, according to the protocols of intensive care,(26) was implemented most often in patients at high risk of death, probably due to their critical condition and the promotion of bed mobilization. The promotion of patient contact with the family via phone calls or video calls was more frequent in the living population. This group, despite communicating better with their relatives, presented delirium more frequently than the deceased (17.7% vs. 7.7%). Despite the promotion of mobilization, an activity directly implemented by nurses in 83.6% of the patients included in the study, the frequency of its implementation depended on the high frequency of pronation, which was performed in prolonged cycles of 12-16 hours. Prolonged cycles of pronation together with other factors, such as hypoperfusion and nutritional failures (probably a late start in the ICU), lead to the appearance of pressure ulcers in 25% of patients, with a higher prevalence in deceased patients (38.7%) with worse critical state, in line with other studies.(20,27,28)

 Oral hygiene, a basic part of nursing care, has been highly recommended in patients in the ICU on mechanical ventilation, since the oral cavity is considered a reservoir for cross-infection for caregivers and one of the routes of entry of microorganisms. In our study, oral hygiene was performed more frequently in living patients than in deceased patients. These results agree with those reported in the study by Kamel *et al.*,(29) in which it was observed that a poor state of oral health, that is, with low hourly cleaning, was related to a greater severity of COVID-19. On the other hand, a high frequency of general bed bathing was maintained to provide comfort to patients. The data of this study give us information on the characteristics, clinical evolution, complications, and nursing interventions that were administered to adult patients hospitalized in four ICUs of Colombian hospitals.

The limitations of the study are related to the possibility of selection biases related to the noninclusion of all patients consecutively. This was because critically ill patients could not sign the informed consent form themselves, and their family members, because they could not enter hospitals during the pandemic, could not authorize entry into the research study. Likewise, it is possible that the biases may have been related to incomplete data that should have been collected retrospectively from the medical records once the patient had died. The observations made in this study correspond to the characteristics of the patients who received care in the four participating institutions and the management protocols of each institution; therefore, the generalization of their results is limited to the observed hospitals.

## Conclusion

Since the beginning of the SARS-CoV-2 pandemic in Colombia and in the rest of the world, nursing personnel in ICUs have had to initiate a process of relearning and skills training for the care of critical patients with ARDS. Although nurses implement a number of interventions derived from medical orders, the progress and recovery of each patient in intensive care depends on the combination of these measures with their own measures within the role that nurses play on an hourly basis. Each intervention depends on an assessment of the health status and the adequate interpretation of the vital signs, from which the results of the administration of medications, respiratory care, positioning, and identification and timely management of complications in the ICU are derived.

 In conclusion, the findings of our study add to the understanding of the behavior and the impact of the COVID-19 pandemic in Colombia, which are very similar to those in the rest of the world. The progress of the pandemic, the rapidity of the transmission of the disease, its evolution, and its impact on certain systems, such as the respiratory system, allowed us to recognize the need to build an appropriate multidisciplinary care plan in intensive care applicable to future epidemics. The behavior of the pandemic, similar to that observed in the rest of the world, in terms of mortality and demographic characterization, comorbidities, ventilatory parameters, and complications allowed us to understand the importance of coordinated multidisciplinary work and to highlight the importance of specific nursing care linked to all structured treatment processes in the ICU. The findings of the study improve our understanding of the pandemic in Colombia and will help in the construction of strategic and curriculum plans for the future management of similar situations.
